# Evidence for the Emergence of New Rice Types of Interspecific Hybrid Origin in West African Farmers' Fields

**DOI:** 10.1371/journal.pone.0007335

**Published:** 2009-10-06

**Authors:** Edwin Nuijten, Robbert van Treuren, Paul C. Struik, Alfred Mokuwa, Florent Okry, Béla Teeken, Paul Richards

**Affiliations:** 1 Technology and Agrarian Development Group, Wageningen University, Wageningen, the Netherlands; 2 Centre for Genetic Resources, the Netherlands, Wageningen University and Research Centre, Wageningen, the Netherlands; 3 Centre for Crop Systems Analysis, Wageningen University, Wageningen, the Netherlands; Cairo University, Egypt

## Abstract

In West Africa two rice species (*Oryza glaberrima* Steud. and *Oryza sativa* L.) co-exist. Although originally it was thought that interspecific hybridization is impossible without biotechnological methods, progenies of hybridization appear to occur in farmer fields.

AFLP analysis was used to assess genetic diversity in West Africa (including the countries The Gambia, Senegal, Guinea Bissau, Guinea Conakry, Sierra Leone, Ghana and Togo) using 315 rice samples morphologically classified prior to analysis. We show evidence for farmer interspecific hybrids of African and Asian rice, resulting in a group of novel genotypes, and identify possible mechanisms for in-field hybridization. Spontaneous back-crossing events play a crucial role, resulting in different groups of genetic diversity in different regions developed by natural and cultural selection, often under adverse conditions. These new groups of genotypes may have potential relevance for exploitation by plant breeders. Future advances in crop development could be achieved through co-operation between scientists and marginalized farmer groups in order to address challenges of rapid adaptation in a world of increasing socio-political and climatic uncertainty.

## Introduction

Rice (*Oryza* spp.) is one of the two most important grain crops worldwide. Its genetic diversity is a factor in securing local and global food security. West Africa is important for genetic diversity of rice, because, uniquely, two species – African rice (*Oryza glaberrima* Steud.) and Asian rice (*Oryza sativa* L.) *–* co-exist within the region. African rice was presumably first cultivated in Mali, Senegal and Guinea Conakry, ±3500 years ago [1], [2]. The history of Asian rice in West Africa is still uncertain, with introduction possible via Arab and/or Portuguese trading networks, ±500–800 years ago. Asian rice has more recently tended to replace African rice, but African rice has persisted or made a modest come-back in some areas, including parts of coastal West Africa.

Several reports claimed that *O. sativa* is completely isolated from *O. glaberrima* by an F1 sterility barrier [3], [4]. Hence, the development of the Nericas (New Rice for Africa) based on the hybridisation of *O. sativa* and *O. glaberrima* was considered a technological breakthrough [5], [6]. However, some scientists suggested that introgression between the two rice species occurs in the field [7], [8]. Based on experiments, Sano [9] argued that pollen flow occurs mainly from *O. sativa* to *O. glaberrima*. Other experimental studies showed that introgression from *O. glaberrima* to *O. sativa* is possible, although at a low frequency [10]–[13]. Artificial backcrosses produced fertile progenies which resembled the parental phenotypes, indicating that under natural conditions it will be difficult to detect hybrid derivatives [9], [14]. This means that, for example, plants belonging to *O. glaberrima* can incorporate *O. sativa* genetic material but remain typically *O. glaberrima* to the eye.

Recent evidence suggests that interspecific hybridization does occur in farmers' fields resulting in new varieties [15]–[18]. Our paper shows that West African farmers have generated their own rices of interspecific background - genetically different from and independent of the scientific initiative leading to Nerica - and suggests possible mechanisms for in-field hybridization behind this major local genetic development, with spontaneous backcrossing playing a crucial role. Our results strongly suggest that interspecific hybridization in West Africa farmers' fields is a recurrent and continuing process, resulting in different groups of genetic diversity in different rice growing areas stimulated by (cultural) differences in selection. Our findings support the hypothesis by Sano et al. [14] that hybridisation followed by backcrossing between *O. sativa* and *O. glaberrima* might lead to the development ‘of new variants not belonging to either of the two species’. These findings might have important implications for understanding crop development and human adaptation. For some time, it has been argued that small-scale farmers in the poorest countries should be consulted about crop improvement, to ensure a better fit between scientific innovation and local food security needs [19]. Now, molecular information is available on the importance of farmer agency during the domestication of rice [20]. We suggest that the current relationship between science and African farmers needs change. Our evidence shows that African farmers are active agents in plant improvement and we suggest that their agency may be taken as a starting point for scientific technology development. New lateral forms of cooperation are required to exploit fully the available genetic diversity of rice.

## Materials and Methods

**Figure 1:**
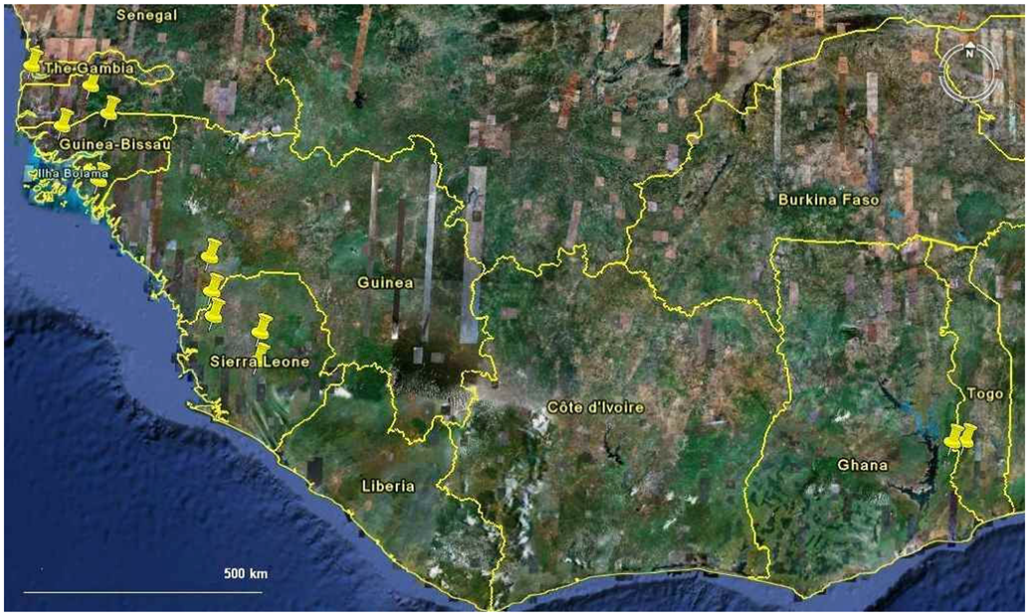
Geographic overview of the West African study area. Pushpins indicate study areas.

We sampled the coastal West African rice belt, including Senegal, The Gambia, Guinea Bissau, Guinea Conakry and Sierra Leone, and the Togo hills rice cultivation outlier in Ghana and Togo (Fig. 1). For demarcation of the upland rice ecology we followed local farmers' definitions. Per country, three or four villages/village clusters were selected, based on ecological and/or cultural contrasts. Per village, as full a set as possible of locally available dryland rice varieties was assembled. Per rice sample, 100–200 panicles were taken at random from the harvest as representative of a variety. Based on farmers' descriptions of the morphological identity of varieties, each rice sample was cleaned carefully. Thus farmer variety samples were morphologically as uniform as formal (released) varieties in the study.

Molecular analysis with AFLP markers, using the *Eco*RI primer E13 in combination with each of the *Mse*I primers M49 or M51, basically followed the procedures described in Nuijten and Van Treuren [16]. AFLP data from 231 collected samples were combined with those of 84 rice samples analysed previously by Nuijten and Van Treuren [16]. A total number of 176 bands was scored, of which 161 were found to be polymorphic. The programme ‘SplitsTree’ was used to visualize phylogenetic relationships between the samples [21] and version 2.2 of the software package ‘Structure’ was used to analyze genetic population structure and to assign samples to populations [22], [23]. To quantify gene variation within groups of samples, Nei's gene diversity (*H*_e_) was calculated [24].

Information about trait and variety preferences, and the origin and spread of varieties, was obtained through quantitative and qualitative interviews with farmers from whom the rice samples were collected (in countries listed above).

Information on morphological features was collected in a field trial carried out in Sierra Leone to characterise morphologically the majority of the materials. The trial design and measurement of the traits followed the procedures described in Nuijten and Van Treuren [16]

### Definitions

Interspecific hybrids: varieties which result from hybridization between *O. sativa* and *O. glaberrima*.

Nerica: improved varieties released by the African Rice Center (formerly WARDA) that result from artificial hybridization between *O. sativa* and *O. glaberrima* followed by two backcrosses to the *O. sativa* parent.

Farmer hybrid: variety which results from spontaneous hybridization between *O. sativa* and *O. glaberrima* followed by backcrossing in farmers' fields and subsequent self-pollination.

Off-type: rice plant with a phenotype distinctive from the sown variety and unknown as a variety (including non-cultivated and ‘lost’ varieties). Off-types can result from mixture, genetic mutation or spontaneous hybridization.

Mixture: a rice stand consisting of various genetically different varieties caused by intentional or unintentional mixing.

## Results

**Figure 2:**
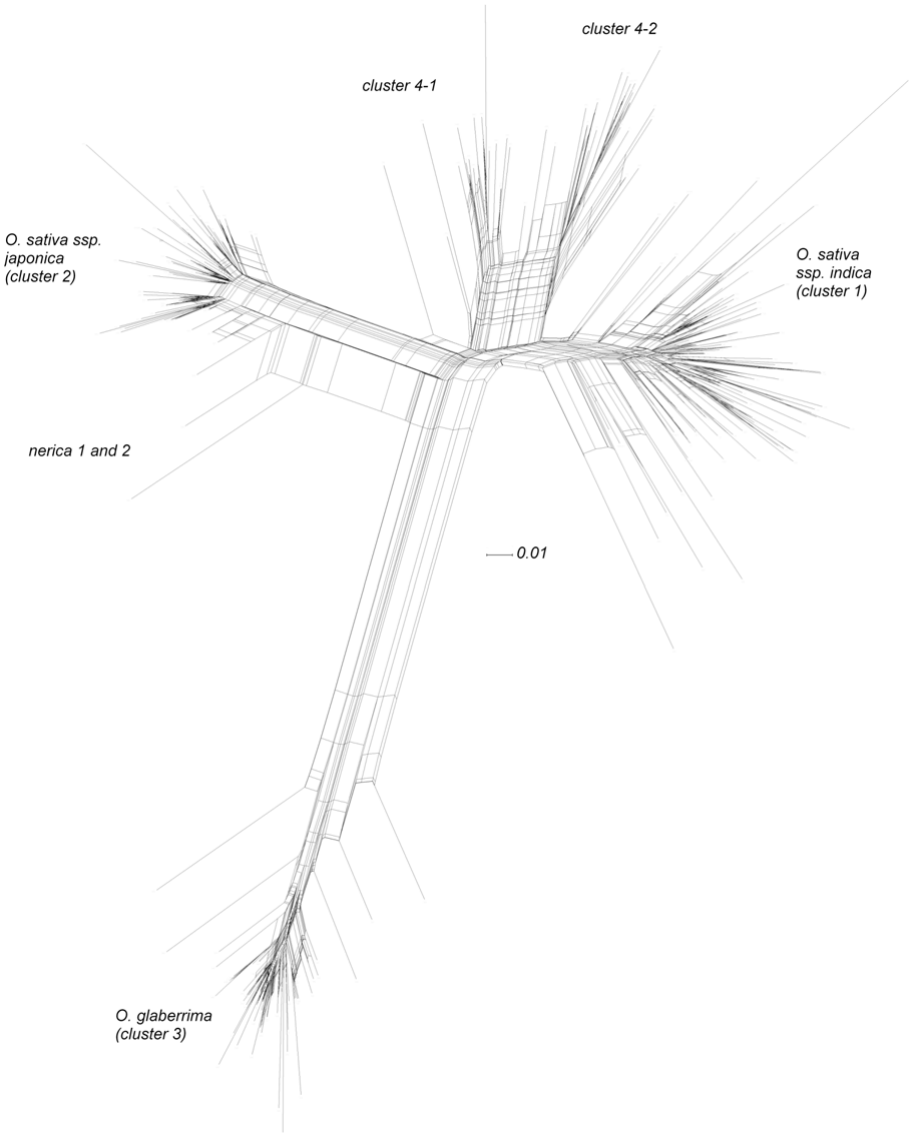
Phylogenetic relationships among the 315 samples studied.

An unrooted phylogenetic network of the 315 rice samples is presented in Fig. 2. As could be expected, *Oryza sativa* ssp. *indica*, *O. sativa* ssp. *japonica* and *O. glaberrima* form three distinct clusters. Nerica varieties of interspecific origin align along the *japonica* axis, with Nerica 1 and 2 facing the *O. glaberrima* branch. In addition to these three clusters, a fourth distinct cluster, consisting of two sub-clusters, was observed, at the junction of the *O. glaberrima-indica-japonica* axes.

Analyses with the software ‘Structure’ showed that the major structure in the data was captured when four populations were assumed. Three of these populations corresponded with *Oryza sativa* ssp. *indica, O. sativa* ssp. *japonica* and *O. glaberrima*, respectively, while the fourth population corresponded with cluster 4 in Fig. 2. Of the 315 materials 285 samples were assigned to a cluster with more than 91% probability. All materials in cluster 4 in Fig. 2 were assigned to cluster 4 with more than 81% probability in Structure, except two varieties from Senegal that were assigned to cluster 4 with 59% and 46% probability.

Prior to the molecular analysis, all varieties collected from farmers were classified as *O. sativa*, *O. glaberrima*, hybrid or unclear. None of the materials assigned to the two *O. sativa* clusters with more than 81% probability were classified as *O. glaberrima* and vice versa (Table 1). The single sample classified as *O. sativa* that was assigned to *O. glaberrima*, and the single sample classified as *O. glaberrima* that was assigned to *O. sativa*, were most likely caused by interchanging of materials during the experiment.

**Table 1:** Presumed taxonomic origin of the 289 farmer varieties in relation to the assignment probabilities to the four observed clusters.

		*O. glaberrima*	Hybrid	Unclear	*O. sativa*
**P (Gla)** *	0.91–1.00	56		6	1
	0.81–0.90	2			
	0.71–0.80				
	0.61–0.70				
	0.51–0.60				
	0.41–0.50				
	0.31–0.40				
	0.21–0.30				
	0.11–0.20		3		
	0.00–0.10	8	16	18	179
**P (Ind)**	0.91–1.00	1	2	6	71
	0.81–0.90			1	3
	0.71–0.80		1		1
	0.61–0.70	1	1		2
	0.51–0.60			1	2
	0.41–0.50				2
	0.31–0.40				
	0.21–0.30				
	0.11–0.20	1	1		
	0.00–0.10	63	14	16	99
**P (Jap)**	0.91–1.00		5	5	70
	0.81–0.90		2		3
	0.71–0.80		1		
	0.61–0.70				
	0.51–0.60				
	0.41–0.50				1
	0.31–0.40				
	0.21–0.30				
	0.11–0.20	1	1		1
	0.00–0.10	65	10	19	105
**P (Cl4)**	0.91–1.00	6	6	5	23
	0.81–0.90		1		2
	0.71–0.80				
	0.61–0.70				
	0.51–0.60				1
	0.41–0.50				2
	0.31–0.40			1	2
	0.21–0.30	1	1		1
	0.11–0.20			1	2
	0.00–0.10	59	11	17	147

* Probabilities of the materials assigned to *O. glaberrima* (Gla), *O. sativa* ssp. *indica* (Ind), *O. sativa* ssp. *japonica* (Jap) and the fourth cluster (Cl4).

**Figure 3:**
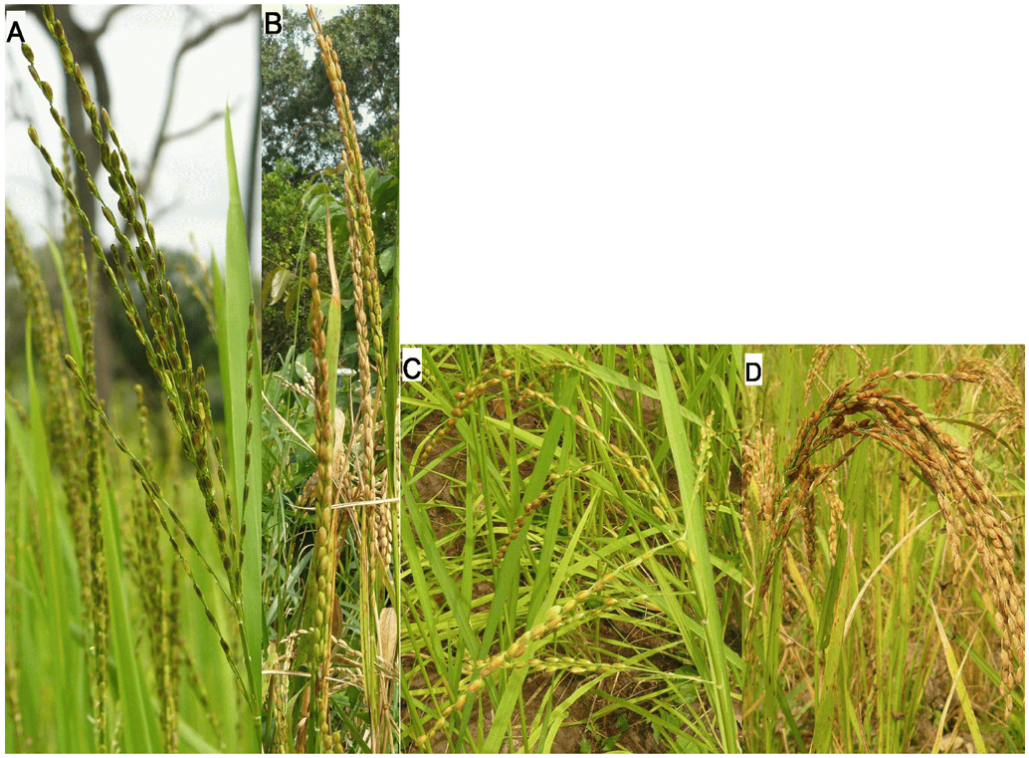
Main panicle types found in this study. Panicle stature of *O. glaberrima* (A), interspecific hybrids from sub-cluster 4-1 with erect (B) and intermediate (C) panicles respectively, and *O. sativa* and interspecific hybrids from sub-cluster 4-2 (D).

Cluster 4 comprised two subclusters (Fig. 2). All varieties in sub-cluster 4-2 had been taxonomically determined as *O. sativa* prior to the molecular study, while cluster 4-1 consisted of samples that had been determined either as *O. sativa*, *O. glaberrima*, hybrid or unclear (Table 2). The main distinctive features between these two sub-clusters were panicle stature at maturity and pericarp (or seed) colour. Sub-cluster 4-1 consisted of varieties with an erect panicle, typical for *O. glaberrima* (Fig. 3), or a semi-erect or slightly drooping panicle, and a red pericarp, except for a single variety from Senegal which had a brown pericarp. Farmers classify particularly the varieties with an erect panicle as *O. glaberrima*, because of the similarity in panicle stature. Farmers do not recognise the varieties of cluster 4 as a separate group. They divide all varieties into two types: those that resemble *O. sativa* and those that resemble *O. glaberrima*. Farmers are not specifically interested in varieties of interspecific origin, but in varieties that perform best under their conditions.

**Table 2:** Presumed taxonomic origin of the farmer hybrid varieties observed in sub-clusters 4-1 and 4-2 in Fig. 2.

Presumed taxonomic origin	Sub-cluster 4-1	Sub-cluster 4-2
*O. sativa*	3	24
*O. glaberrima*	6	0
Hybrid	7	0
Unclear	5	0
Total	21	24

The three varieties in sub-cluster 4-1 that were classified as *O. sativa* had semi-droopy panicles which made them less distinctive from *O. sativa*. Sub-cluster 4-2 consisted of varieties in which panicles were predominantly strongly drooping, similar to *O. sativa*, and in which the pericarp colour varied from white to brown (90% of the varieties had a brown pericarp colour). Except for pericarp colour, the varieties in sub-cluster 4-2 did not have any clearly distinctive morphological features from *O. sativa* varieties (Table 3). Detailed morphological analysis of some varieties belonging to sub-cluster 4-2 in 2002 showed that when characteristics were aggregated in a Principal Component Analysis these farmer varieties were different from *O. sativa* ssp. *indica* and *O. sativa* ssp. *japonica* [16].

**Table 3:** Main distinctive morphological features of 12 varieties from cluster 4*.

Variety name	Country	Sub-cluster	Panicle attitude	Ligule shape	Pericarp colour	Days to 80% flowering
Tebeleh	Sierra Leone	4-1	erect	pointed, long	red	105.8
Pa DC	Sierra Leone	4-1	erect	pointed, long	red	103.8
Pa Trimont	Sierra Leone	4-1	semi-droopy	pointed, long	red	92.5
Wonyonwonyon yi	Guinea Conakry	4-1	semi-droopy	pointed, long	red	96.3
Untufa	Guinea Bissau	4-1	erect	pointed, long	red	98.0
Dissi	Guinea Bissau	4-1	erect	pointed, long	red	104.0
Mani Konsunkuto	Guinea Bissau	4-2	strongly droopy	pointed, long	brown	87.5
Kolosar, Mani Wulendingo	Guinea Bissau	4-2	strongly droopy	pointed, long	white	91.8
Mani Wulengo	Gambia	4-2	strongly droopy	pointed, long	brown	88.0
Binta Sambou**	Gambia	4-2	strongly droopy	pointed, long	light brown	103.3
Ablie Mano	Senegal	4-2	droopy	pointed, long	brown	89.5
Madina Wulengo	Senegal	4-2	strongly droopy	pointed, long	brown	90.8

* Varieties of *O. glaberrima* included in this study had erect panicle, round short ligule and red pericarp colour. Varieties of *O. sativa* ssp. included in this study had strongly droopy panicle, pointed medium to long ligule, and white or red pericarp colour.

** In The Gambia Binta Sambou flowers only a few days later than Ablie Mano.

Genetic diversity within groups (*H*_e_) was calculated for each of the four clusters. For this purpose an assignment probability of 91% was used as cut-off point to define the four clusters. The *H*_e_ value for cluster 4 was highest (0.098; n = 40) followed closely by the *H*_e_ value for the *O. sativa* ssp. *indica* group (0.089; n = 92). Relatively low values were observed for the *O. sativa* ssp. *japonica* group (0.045; n = 87) and the *O. glaberrima* group (0.034, n = 66).

**Figure 4:**
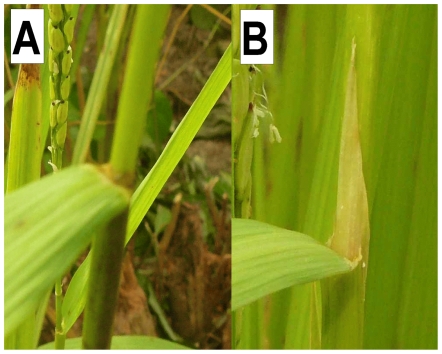
Main ligule shapes found in this study. Ligule shape of *O. glaberrima* (A: small, rounded) and *O. sativa* and interspecific hybrids from cluster 4 (B: long, pointed).

Varieties in sub-cluster 4-1 not only displayed characteristics typical of *O. glaberrima*, such as the easily observable erect panicle stature (Fig. 3), but also characteristics of *O. sativa*, such as the long, pointed ligule typical of *O. sativa* (Fig. 4), a less conspicuous feature. The only explanation for this new morphotype is interspecific hybridization between *O. sativa* and *O. glaberrima*. This was supported by the molecular data, separating cluster 4 from *O. sativa* ssp. and *O. glaberrima,* and showing large within-group diversity.

Cluster 4 consisted of a considerable number of different farmer interspecific hybrids originating from the Upper West African coastal rice belt (Table 4). None of the modern varieties and none of the samples collected in Ghana and Togo were found in cluster 4 in Fig. 2, nor were any of these samples assigned to cluster 4 in Table 4 with more than 40% probability. Thirty samples - originating from almost all countries, and including two modern varieties - were assigned with less than 91% probability to one cluster. No samples from Togo were assigned with less than 91% probability to one cluster. Although no samples from Ghana were assigned to cluster 4, five samples were assigned with high probabilities to two clusters. These samples may require further study to know whether they have an interspecific background. But we cannot assume that all such materials have an interspecific nature since one variety from IRRI was assigned to the *O. sativa* ssp. *indica* group with 76% probability (Table S1). Likewise, existence of samples with a very high assignment percentage probability does not rule out an interspecific origin. For example, WAB 450-I-B-P-105-HB, a Nerica that was never officially released was assigned with 100% probability to the *O. sativa* ssp. *japonica* group.

**Table 4:** Number of farmer varieties, modern varieties and (semi-) wild relatives assigned by the software ‘Structure’ to the four observed clusters.

		The Gambia	Senegal	Guinea Bissau	Guinea Conakry	Sierra Leone	Ghana	Togo	Modern	(Semi) wild	Total
**P (Gla)** *	0.91–1.00	4	3	4	25	8	10	9		3	66
	0.81–0.90		1	1						1	3
	0.71–0.80									1	1
	0.61–0.70										
	0.51–0.60										
	0.41–0.50										
	0.31–0.40										
	0.21–0.30										
	0.11–0.20	2					1				3
	0.00–0.10	53	18	36	21	52	35	6	21		242
**P (Ind)**	0.91–1.00	23	7	5	14	8	20	3	12		92
	0.81–0.90	1			1	1	1				4
	0.71–0.80		1				1		1		3
	0.61–0.70	2			1		1				4
	0.51–0.60		1	1			1				3
	0.41–0.50		1				1				2
	0.31–0.40										
	0.21–0.30										
	0.11–0.20		1		1					1	3
	0.00–0.10	33	11	35	29	51	21	12	8	4	204
**P (Jap)**	0.91–1.00	18		18	2	29	10	3	7		87
	0.81–0.90	1		2	1		1				5
	0.71–0.80	1							1		2
	0.61–0.70										
	0.51–0.60										
	0.41–0.50						1				1
	0.31–0.40										
	0.21–0.30										
	0.11–0.20	1	1	1							3
	0.00–0.10	38	21	20	43	31	34	12	13	5	217
**P (Cl4)**	0.91–1.00	8	7	10	2	13					40
	0.81–0.90	1	1			1					3
	0.71–0.80										
	0.61–0.70										
	0.51–0.60		1								1
	0.41–0.50		1	1							2
	0.31–0.40	1					2				3
	0.21–0.30		1		1		1		1		4
	0.11–0.20	1				1	1		1		4
	0.00–0.10	48	11	30	43	45	42	15	19	5	258

Data for the farmer varieties are presented separately per country of origin.

* Probabilities of the materials assigned to *O. glaberrima* (Gla), *O. sativa* ssp. *indica* (Ind), *O. sativa* ssp. *japonica* (Jap) and the fourth cluster (Cl4).

To a certain extent, the sub-clusters relate to the countries of collection and local seed colour preferences. The varieties in sub-cluster 4-1 originate from Guinea Bissau (4), Guinea Conakry (2), Senegal (1) and Sierra Leone (14), while the varieties in sub-cluster 4-2 are from The Gambia (9), Guinea Bissau (6) and Senegal (9). Whereas in Guinea Conakry and Sierra Leone farmers commonly cultivate red rice (both African and Asian rice), farmers in The Gambia, Senegal and northern Guinea Bissau predominantly cultivate white rice. Southern Guinea Bissau occupies an intermediate position, as red rice is still cultivated but farmers strongly prefer white rice.

## Discussion

### Development of interspecific hybrid varieties

The molecular data showed that cluster 4 is more closely related to *O. sativa* than to *O. glaberrima*. This can be explained by the following scenario for the development of interspecific hybrids in farmer fields. The progeny of an F1-hybrid between *O. sativa* and *O. glaberrima* can maintain itself in the gene pool only through backcrossing to either species (*O. sativa* or *O. glaberrima*), because of a high level of sterility of the F1-hybrid. Farmers do not harvest the panicles of an F1-hybrid because (almost) all grains are empty. Hybrids as such are not maintained in a plant population. The event of a flower being pollinated by pollen of the other rice species is not observable. A panicle that carries one seed which is the result of pollination by the other species (and 200 by self-pollination) looks normal. If that panicle is selected for sowing seed, the seed that is produced by the flower pollinated by the other species is sown in the rice field, germinates and produces a hybrid plant. Only after grain filling (usually at harvesting time) can a farmer recognize this plant as an interspecific hybrid because it does not carry any seed and therefore he/she will not harvest it. Backcrossing is the only way for the genes of a hybrid to be incorporated into a new genotype. From this point two sub-scenarios are possible. The first sub-scenario is that a hybrid plant is pollinated by surrounding normal plants and the few seeds produced by the hybrid remain in the field, germinating next season, then to be pollinated by surrounding normal plants, after which fertility is restored and the offspring may be harvested by farmers. This scenario was also suggested by Sano et al. [14]. For this scenario to be possible a farmer needs to crop the same field to rice for at least three consecutive growing seasons, as sometimes happens where land is initially fertile and where abandoned plots are then cleared for re-use by members of a household with low labour capacity, such as widows. Work on Nerica [5] and speciation in rice [14] suggests that two backcrosses are sufficient to obtain ‘offspring’ with good fertility. The second sub-scenario is that during flowering the F1-hybrid may pollinate the surrounding normal plants. A panicle of a normal plant in which one flower is pollinated by the hybrid looks normal and may be included in the seed for next season. Two such backcrossing events to *O. sativa* or *O. glaberrima*, and subsequent replanting of the progeny by farmers should also lead to fertile offspring, given enough time and opportunities. Subsequently, off-types of interspecific origin showing potential may be selected by farmers to be tested, multiplied and grown as new varieties. If other farmers show an interest in such a new variety, it may spread over a wider region. The whole process of the development of interspecific hybrid varieties is a combination of a random process of cross-pollination and backcrossing, followed by a selection process of those off-types that show most potential as new varieties by farmers.

Field studies suggested that introgression can occur in both directions (from *O. glaberrima* to *O. sativa* and vice versa) [7], [8], although some experimental studies have indicated that introgression from *O. sativa* to *O. glaberrima* occurs more often than introgression in the opposite direction [11], [12], as confirmed by field observations in 2002 by Nuijten [25]. Artificial backcrosses produced fertile progenies which resembled the parental phenotypes, indicating that under natural conditions it is difficult to detect hybrid derivatives [9], [14]. Given that the hybrid group (cluster 4) is closer to *O. sativa* than to *O. glaberrima*, successful backcrossing events in the field to *O. sativa* might be more likely than to *O. glaberrima*. According to Sano [9] the combination of nuclear DNA of *O. glaberrima* with cytoplasmic DNA of *O. sativa* always results in cytoplasmic male sterility. This suggests that the farmer hybrids may be the result of backcrossing to *O. sativa* and carry a combination of cytoplasmic DNA of *O. glaberrima* with nuclear DNA mainly from *O. sativa*. Chloroplast DNA analysis may give more conclusive information on whether the farmer hybrids result from *O. glaberrima* × *O. sativa* hybrids or *O. sativa* × *O. glaberrima* hybrids [26], [27]. These results may also clarify which scenario of backcrossing in farmer fields led to the development of the farmer hybrids. But it should also be noted that in both species varieties may exist that are able to overcome the sterility system - so-called Wide Compatibility Varieties [11].

Rice hybridization in farmer's fields may occur when *O. glaberrima* and *O. sativa* flower side by side. There are various scenarios to explain this co-occurrence at field level. The first possibility is the deliberate sowing of mixtures, which has been reported for several localities in the upper West African coastal zone [15], [21], [28]. The second, perhaps more common, possibility is the non-deliberate mixing of *O. glaberrima* within *O. sativa* seed stocks.

Roguing off-types requires skill and effort, and is sometimes neglected due to pressure to harvest the crop quickly, resulting in contamination of *O. sativa* seed batches with *O. glaberrima* seeds. Seed contamination can also reflect indebtedness, since farmers harvesting seed intended for loaning to poorer farmers rarely bother to rogue the material [29]. Because the separation of seed types after threshing is a much harder task than panicle roguing at harvest, contamination of *O. sativa* seed batches with *O. glaberrima* may be as high as 30%. These figures boost chances of spontaneous interspecific hybridization on the farms where seed has been loaned.

Another non-intentional factor is the presence of weedy rice types intermediate between wild African rice (*O. barthii*) and *O. glaberrima* in farmers' fields. Gene flow between weedy types and cultivated Asian rice may also result in some in-field interspecific hybridization. Weedy rice types like “ngewobei” and “ngafabei” (as named by Mende-speaking farmers in central Sierra Leone) may be the result of interspecific hybridization between *O. barthii* and *O. sativa* (Table S1). Such weedy types may provide a bridge between wild and cultivated species for breeders to transfer useful characteristics from wild to cultivated rice.

### Time depth of farmer hybrid-derived rices – historical evidence

Given the release of hybrid-derived interspecific rice varieties in the Nerica series from WARDA (Africa Rice Center) in the late 1990s it is appropriate to provide evidence that the farmer intermediate types analysed in this paper pre-date the Nerica releases. Rice varieties with the name elements “ three month” and “disi” (also written as “DC”) and the same morphological features as the collected varieties with the same name elements belonging to cluster 4-1 were collected by Richards [30] and Jusu [15] in Sierra Leone in 1987–88 and 1995–96, respectively.

Farmers from Guinea Bissau provided the following information in the present study. The interspecific farmer hybrids belonging to cluster 4-1 collected in northern Guinea Bissau were reportedly cultivated before 1940. How much earlier they were cultivated is not clear, since precise data from before 1940 are largely absent. Some farmers considered them to have always been there. This gains some support from some of the names. In northern Guinea Bissau farmers referred to these varieties by names also used for *O. glaberrima*, such as “jangjango”, “untufa”, and “wansarang”. “Jangjango” specifically refers to the upright panicle typical of *O. glaberrima*. The meaning of the variety name “untufa” is ‘rice from here’ because it is considered ancient, implying farmers think it is *O. glaberrima*, the rice originally domesticated in West Africa.

The origin of many varieties from cluster 4-2, such as “mani wulengo”, “mani wulendingo”, “mani konsonkuto”, “ablie mano”, collected in The Gambia, Senegal and Guinea Bissau can be traced back to northern Guinea Bissau. One variety in The Gambia, “binta sambou”, was developed from an off-type found in a field of “ablie mano” around 1990. Except for the variety “binta sambou” farmers could not pinpoint place or time of origin. In one village, Pantufa, in northern Guinea Bissau farmers indicated that varieties such as “mani wulengo”, “mani konsonkuto”, “mani wulendingo” and “ablie mano” were cultivated before 1940.

The information available so far suggests the countries where the interspecific farmer varieties were first cultivated were Sierra Leone and Guinea Bissau. No precise dates of origin can be specified, but the aforementioned data suggest that some existed for more than half a century, and thus long before the first release of Nerica varieties.

### Spread of interspecific farmer hybrids

Adversity such as war and drought appear to have favoured the selection and spread of spontaneous interspecific rice hybrids among West African farmers. War has forced some farmers into intensively farmed pockets of land without access to fertilizers. Farmer hybrids appear to share the adaptation to poor soils of the *O. glaberrima* parent. Parts of the war zone in Sierra Leone, cut off from aid assistance over several years, appeared to be mainly growing interspecific hybrid varieties (or pure *glaberrimas*) in the period immediately after fighting ceased [31]. Farmers noted that war reduced the amount of time available for clearing of forest, weeding and careful harvesting new fields, since civilians were reluctant to linger for fear of encountering fighters. In other cases (e.g. as a result of war in Guinea-Bissau and southern Senegal) they fled across borders, taking their hardy varieties with them. Farmer hybrids are particularly frequent in our samples from southern Senegal, Guinea-Bissau and Sierra Leone (Table 4) − all regions affected by recent episodes of armed conflict.

In Senegal and The Gambia the farmer hybrids have probably helped farmers to cope with climatic fluctuation. The farmer hybrids (belonging to sub-cluster 4-2) collected in these two countries tend to flower about one week earlier than the farmer hybrids (belonging to sub-cluster 4-1) collected in Sierra Leone (Table 3). Senegal and The Gambia have been badly affected by drought in recent times. In addition, both countries have faced increased demographic pressure, exacerbated by armed conflict in southern Senegal and Guinea Bissau. Farmer hybrids may embody considerable adaptive plasticity to suboptimal farming conditions associated with such difficulties.

An important reason why in Senegal and The Gambia farmers mainly grow farmer hybrids belonging to sub-cluster 4-2 is that in these two countries farmers do not like a red pericarp colour (the variety belonging to sub-cluster 4-1 and cultivated in Senegal does not have a red pericarp). In addition, some farmers mentioned they do not like an erect panicle when mature. In Sierra Leone and Guinea Conakry the farmer hybrids found belonged to sub-cluster 4-1. In these two countries farmers prefer a red pericarp colour because they claim it is related to slow digestion. Also they do not consider an erect panicle a negative trait. These two traits are the main traits that differentiate sub-clusters 4-1 and 4-2. Both can be considered polygenic traits which may explain why farmer selection practices have resulted in large genetic differences between the two sub-clusters, as is shown by the molecular data. Given the different ecological and climatic conditions in the region, the outcome of farmer selection for traits such as panicle length, tillering, plant height, yield, taste, swelling, and ease of threshing may possibly have contributed to the genetic differences between sub-clusters 4-1 and 4-2.

### Why are interspecific farmer hybrids absent or rare in Ghana and Togo?

Farmer interspecific hybrids are less frequent or absent in our samples from Ghana and Togo (Togo Hills), an important region of co-occurrence of *O. glaberrima* and *O. sativa*. Conditions in the Togo Hills may be less favourable to in-field interspecific hybridization due to cultural and geographical factors. The cultural significance of African rice seems to limit the amount of farmer hybridization on the Ghana side of the Togo Hills. Rice cultivators in eastern Ghana grow *O. sativa* mainly as a commercial crop under relatively favourable conditions. These farmers maintain a strong interest in African rice, but for cultural reasons. African rice is prominent in traditional ceremonies and as an ethnic marker [32]. In such circumstances, a hybrid would be less suited because of its blurred morphology. Farmers in Togo (the Danyi plateau) grow African rice at higher altitudes, while *O. sativa* is planted at lower altitudes. This imposes a geographical barrier to interspecific hybridization.

### Concluding remarks

Our results strongly suggest that interspecific hybridization in West African farmers' fields is a recurrent and continuing process, with spontaneous back-crossing events playing a crucial role, resulting in different groups of genetic diversity in different rice growing areas stimulated by differences in selection criteria and selection environments. This clear evidence for the emergence of farmer hybrids of African and Asian rice in West Africa has important implications for understanding crop development and human adaptation. Whether and how such hybridisation and backcrossing events have occurred for other crops may be a useful question to pursue, to achieve a better understanding of crop development and diversity. For example, it may help to identify the most plausible scenario for the development of maize (*Zea mays* L.). Our findings also suggest that adversity, such as dislocation by armed conflict and climatic change, has not hindered, and may have accelerated the rate at which interspecific hybrid rice varieties have spread [31]. Farmer interspecific hybrids of rice may complement those recently developed by formal scientific research. This points to potential value in linking science and local technology development by marginalized groups, better to address challenges of rapid adaptation in a world of increased socio-political and climatic uncertainty.

## Supporting Information

Table S1 Overview of the 315 investigated rice samples and their assignment to the four observed clusters by the software Structure.
(0.64 MB DOC)
