# Supplementary material for: Evidence for the Emergence of New Rice Types of Interspecific Hybrid Origin in West African Farmers' Fields
Source: PLoS One. 2009 Oct 6;4(10):e7335. doi: 10.1371/journal.pone.0007335 (PMC2752159; doi:10.1371/journal.pone.0007335)
Supplement: Table S1 — Overview of the 315 investigated rice samples and their assignment to the four observed clusters by the software Structure. (0.64 MB DOC) [file pone.0007335.s001.doc]

| **Variety name** | **Origin** | **Taxonomy** | **P (Gla)** | **P (Ind)** | **P (Jap)** | **P (Cl4)** |
| --- | --- | --- | --- | --- | --- | --- |
|  |  |  |  |  |  |  |
| **A. Farmer varieties** |  |  |  |  |  |  |
| Kaomo black | Ghana | O. glaberrima | 1.00 | 0.00 | 0.00 | 0.00 |
| Kaomo black (with awns) | Ghana | O. glaberrima | 1.00 | 0.00 | 0.00 | 0.00 |
| Kaomo krukutuwa | Ghana | O. glaberrima | 1.00 | 0.00 | 0.00 | 0.00 |
| Kaomo krukutuwa signaweh | Ghana | O. glaberrima | 1.00 | 0.00 | 0.00 | 0.00 |
| Kaomo signaweh | Ghana | O. glaberrima | 1.00 | 0.00 | 0.00 | 0.00 |
| Kaomo signaweh black | Ghana | O. glaberrima | 1.00 | 0.00 | 0.00 | 0.00 |
| Kaomo white | Ghana | O. glaberrima | 1.00 | 0.00 | 0.00 | 0.00 |
| Jangjango | Guinea Bissau | O. glaberrima | 1.00 | 0.00 | 0.00 | 0.00 |
| Jangjango | Guinea Bissau | O. glaberrima | 1.00 | 0.00 | 0.00 | 0.00 |
| Kurekimbeli | Guinea Bissau | O. glaberrima | 1.00 | 0.00 | 0.00 | 0.00 |
| Uassolondji | Guinea Bissau | O. glaberrima | 1.00 | 0.00 | 0.00 | 0.00 |
| Dixi Wansan Lot 1 | Guinea Conakry | O. glaberrima | 1.00 | 0.00 | 0.00 | 0.00 |
| Saali Fire | Guinea Conakry | O. glaberrima | 1.00 | 0.00 | 0.00 | 0.00 |
| Saali Fore | Guinea Conakry | O. glaberrima | 1.00 | 0.00 | 0.00 | 0.00 |
| Saali Fore | Guinea Conakry | O. glaberrima | 1.00 | 0.00 | 0.00 | 0.00 |
| Saali Fore | Guinea Conakry | O. glaberrima | 1.00 | 0.00 | 0.00 | 0.00 |
| Saali Fore | Guinea Conakry | O. glaberrima | 1.00 | 0.00 | 0.00 | 0.00 |
| Saali Fore | Guinea Conakry | O. glaberrima | 1.00 | 0.00 | 0.00 | 0.00 |
| Siiga? | Guinea Conakry | O. glaberrima | 1.00 | 0.00 | 0.00 | 0.00 |
| Tombo Bokary | Guinea Conakry | O. glaberrima | 1.00 | 0.00 | 0.00 | 0.00 |
| Tombo Bokary | Guinea Conakry | O. glaberrima | 1.00 | 0.00 | 0.00 | 0.00 |
| Tombo Bokary | Guinea Conakry | O. glaberrima | 1.00 | 0.00 | 0.00 | 0.00 |
| Tombo Bokary | Guinea Conakry | O. glaberrima | 1.00 | 0.00 | 0.00 | 0.00 |
| Tombo Bokary | Guinea Conakry | O. glaberrima | 1.00 | 0.00 | 0.00 | 0.00 |
| Tombo Bokary | Guinea Conakry | O. glaberrima | 1.00 | 0.00 | 0.00 | 0.00 |
| Mani Musoo | Senegal | O. glaberrima | 1.00 | 0.00 | 0.00 | 0.00 |
| Mani Musoo | Senegal | O. glaberrima | 1.00 | 0.00 | 0.00 | 0.00 |
| Damba | Sierra Leone | O. glaberrima | 1.00 | 0.00 | 0.00 | 0.00 |
| Saliforeh | Sierra Leone | O. glaberrima | 1.00 | 0.00 | 0.00 | 0.00 |
| Mani Ba | The Gambia | O. glaberrima | 1.00 | 0.00 | 0.00 | 0.00 |
| Mani Ba | The Gambia | O. glaberrima | 1.00 | 0.00 | 0.00 | 0.00 |
| Mani Ba | The Gambia | O. glaberrima | 1.00 | 0.00 | 0.00 | 0.00 |
| Mani Ba | The Gambia | O. glaberrima | 1.00 | 0.00 | 0.00 | 0.00 |
| Awinto blanc | Togo | O. glaberrima | 1.00 | 0.00 | 0.00 | 0.00 |
| Awinto yibo | Togo | O. glaberrima | 1.00 | 0.00 | 0.00 | 0.00 |
| Danyi moli | Togo | O. glaberrima | 1.00 | 0.00 | 0.00 | 0.00 |
| Danyi moli | Togo | O. glaberrima | 1.00 | 0.00 | 0.00 | 0.00 |
| Kpakpalipke | Togo | O. glaberrima | 1.00 | 0.00 | 0.00 | 0.00 |
| Xleti etoh (three months) | Togo | O. glaberrima | 1.00 | 0.00 | 0.00 | 0.00 |
| Xleti eve | Togo | O. glaberrima | 1.00 | 0.00 | 0.00 | 0.00 |
| Yibo riz | Togo | O. glaberrima | 1.00 | 0.00 | 0.00 | 0.00 |
| Kaomo signaweh white | Ghana | O. glaberrima | 0.99 | 0.00 | 0.00 | 0.00 |
| Saali Koute | Guinea Conakry | O. glaberrima | 0.99 | 0.01 | 0.00 | 0.00 |
| Tombo Bokary | Guinea Conakry | O. glaberrima | 0.99 | 0.00 | 0.00 | 0.00 |
| Mani Musoo | Senegal | O. glaberrima | 0.99 | 0.01 | 0.00 | 0.00 |
| Sanganyaa | Sierra Leone | O. glaberrima | 0.99 | 0.00 | 0.00 | 0.01 |
| Dixi Wansan Lot 2 | Guinea Conakry | O. glaberrima | 0.98 | 0.00 | 0.01 | 0.00 |
| Safaary | Guinea Conakry | O. glaberrima | 0.98 | 0.00 | 0.00 | 0.01 |
| Siiga | Guinea Conakry | O. glaberrima | 0.98 | 0.01 | 0.01 | 0.01 |
| Tombo Bokary | Guinea Conakry | O. glaberrima | 0.98 | 0.00 | 0.00 | 0.02 |
| Gbankeyi | Guinea Conakry | O. glaberrima | 0.97 | 0.01 | 0.00 | 0.02 |
| Safaary | Guinea Conakry | O. glaberrima | 0.96 | 0.01 | 0.02 | 0.01 |
| Awinto blanc | Togo | O. glaberrima | 0.93 | 0.03 | 0.00 | 0.03 |
| Maalay | Sierra Leone | O. glaberrima | 0.92 | 0.03 | 0.03 | 0.02 |
| Saali Fore | Guinea Conakry | O. glaberrima | 0.91 | 0.06 | 0.02 | 0.01 |
| Saali Fore | Guinea Conakry | O. glaberrima | 0.91 | 0.00 | 0.02 | 0.07 |
| Mani Musoo | Senegal | O. glaberrima | 0.82 | 0.14 | 0.00 | 0.04 |
| Wansarang | Guinea Bissau | O. glaberrima | 0.81 | 0.02 | 0.14 | 0.02 |
| Siiga | Guinea Conakry | O. glaberrima | 0.01 | 0.98 | 0.00 | 0.01 |
| Dalifode | Guinea Conakry | O. glaberrima | 0.10 | 0.67 | 0.01 | 0.22 |
| Trimont (white) | Sierra Leone | O. glaberrima | 0.00 | 0.00 | 0.00 | 1.00 |
| Pa Trimont | Sierra Leone | O. glaberrima | 0.00 | 0.00 | 0.00 | 0.99 |
| Painy-pain | Sierra Leone | O. glaberrima | 0.00 | 0.00 | 0.00 | 0.99 |
| Pindie | Sierra Leone | O. glaberrima | 0.00 | 0.00 | 0.00 | 0.99 |
| Pa Trimont (red) | Sierra Leone | O. glaberrima | 0.01 | 0.01 | 0.00 | 0.98 |
| Saliforeh | Sierra Leone | O. glaberrima | 0.00 | 0.01 | 0.03 | 0.96 |
| Samba | Guinea Conakry | O. sativa | 0.97 | 0.01 | 0.02 | 0.01 |
| Adeisi | Ghana | O. sativa | 0.00 | 1.00 | 0.00 | 0.00 |
| Akpasseh | Ghana | O. sativa | 0.00 | 1.00 | 0.00 | 0.00 |
| Red saka | Ghana | O. sativa | 0.00 | 1.00 | 0.00 | 0.00 |
| Zomojo | Ghana | O. sativa | 0.00 | 1.00 | 0.00 | 0.00 |
| Bissau | Guinea Bissau | O. sativa | 0.00 | 1.00 | 0.00 | 0.00 |
| Sajar | Guinea Bissau | O. sativa | 0.00 | 1.00 | 0.00 | 0.00 |
| Kaniya | Guinea Conakry | O. sativa | 0.00 | 1.00 | 0.00 | 0.00 |
| Momodou male | Guinea Conakry | O. sativa | 0.00 | 1.00 | 0.00 | 0.00 |
| Saidou fire (red grain) | Guinea Conakry | O. sativa | 0.00 | 1.00 | 0.00 | 0.00 |
| Soumaila | Guinea Conakry | O. sativa | 0.00 | 1.00 | 0.00 | 0.00 |
| Ablie Koyo | Senegal | O. sativa | 0.00 | 1.00 | 0.00 | 0.00 |
| Fadass | Senegal | O. sativa | 0.00 | 1.00 | 0.00 | 0.00 |
| Kuboni | Senegal | O. sativa | 0.00 | 1.00 | 0.00 | 0.00 |
| Madina Koyo | Senegal | O. sativa | 0.00 | 1.00 | 0.00 | 0.00 |
| Buttercup | Sierra Leone | O. sativa | 0.00 | 1.00 | 0.00 | 0.00 |
| Yainky-Yanka | Sierra Leone | O. sativa | 0.00 | 1.00 | 0.00 | 0.00 |
| Akacha | The Gambia | O. sativa | 0.00 | 1.00 | 0.00 | 0.00 |
| Barafita koyo | The Gambia | O. sativa | 0.00 | 1.00 | 0.00 | 0.00 |
| Baraso | The Gambia | O. sativa | 0.00 | 1.00 | 0.00 | 0.00 |
| Bendou | The Gambia | O. sativa | 0.00 | 1.00 | 0.00 | 0.00 |
| Chinese short | The Gambia | O. sativa | 0.00 | 1.00 | 0.00 | 0.00 |
| Derisa Mano | The Gambia | O. sativa | 0.00 | 1.00 | 0.00 | 0.00 |
| Foni Mano | The Gambia | O. sativa | 0.00 | 1.00 | 0.00 | 0.00 |
| Off-type (in Binta Sambou) | The Gambia | O. sativa | 0.00 | 1.00 | 0.00 | 0.00 |
| Peking | The Gambia | O. sativa | 0.00 | 1.00 | 0.00 | 0.00 |
| Peking | The Gambia | O. sativa | 0.00 | 1.00 | 0.00 | 0.00 |
| Tensi | The Gambia | O. sativa | 0.00 | 1.00 | 0.00 | 0.00 |
| Tombom | The Gambia | O. sativa | 0.00 | 1.00 | 0.00 | 0.00 |
| Adeta red rice | Ghana | O. sativa | 0.00 | 0.99 | 0.00 | 0.01 |
| Awonyo (two months) | Ghana | O. sativa | 0.00 | 0.99 | 0.01 | 0.01 |
| Bouake | Ghana | O. sativa | 0.00 | 0.99 | 0.00 | 0.01 |
| James rice | Ghana | O. sativa | 0.00 | 0.99 | 0.00 | 0.01 |
| Red saka | Ghana | O. sativa | 0.00 | 0.99 | 0.00 | 0.00 |
| Red saka (off-type?) | Ghana | O. sativa | 0.00 | 0.99 | 0.00 | 0.01 |
| Red variety | Ghana | O. sativa | 0.00 | 0.99 | 0.00 | 0.00 |
| Aninha de lugar | Guinea Bissau | O. sativa | 0.00 | 0.99 | 0.00 | 0.01 |
| Wankarang | Guinea Bissau | O. sativa | 0.00 | 0.99 | 0.00 | 0.00 |
| Saidou Fire | Guinea Conakry | O. sativa | 0.00 | 0.99 | 0.00 | 0.00 |
| Saidou Fire | Guinea Conakry | O. sativa | 0.00 | 0.99 | 0.00 | 0.01 |
| Saidou Gbeeli | Guinea Conakry | O. sativa | 0.00 | 0.99 | 0.00 | 0.00 |
| Saidou Gbeeli | Guinea Conakry | O. sativa | 0.00 | 0.99 | 0.00 | 0.00 |
| Jina Mano | Senegal | O. sativa | 0.00 | 0.99 | 0.00 | 0.00 |
| Kuboni Juuno | Senegal | O. sativa | 0.00 | 0.99 | 0.00 | 0.00 |
| Rok31 | Sierra Leone | O. sativa | 0.00 | 0.99 | 0.00 | 0.00 |
| Bonti | The Gambia | O. sativa | 0.00 | 0.99 | 0.00 | 0.01 |
| Kadi Dabo | The Gambia | O. sativa | 0.00 | 0.99 | 0.00 | 0.00 |
| Mani Suntungo-1 | The Gambia | O. sativa | 0.00 | 0.99 | 0.00 | 0.01 |
| Mani Suntungo-2 | The Gambia | O. sativa | 0.00 | 0.99 | 0.00 | 0.00 |
| Muso Noringo | The Gambia | O. sativa | 0.00 | 0.99 | 0.00 | 0.01 |
| Peking | The Gambia | O. sativa | 0.00 | 0.99 | 0.00 | 0.01 |
| Sainey Kolly | The Gambia | O. sativa | 0.00 | 0.99 | 0.01 | 0.01 |
| Teiba | The Gambia | O. sativa | 0.00 | 0.99 | 0.00 | 0.01 |
| Awuie red | Togo | O. sativa | 0.00 | 0.99 | 0.00 | 0.00 |
| Awuie white | Togo | O. sativa | 0.00 | 0.99 | 0.00 | 0.01 |
| White saka | Ghana | O. sativa | 0.00 | 0.98 | 0.00 | 0.02 |
| Sambaconcon | Guinea Bissau | O. sativa | 0.02 | 0.98 | 0.00 | 0.00 |
| CK 21 | Guinea Conakry | O. sativa | 0.01 | 0.98 | 0.01 | 0.01 |
| Pode 1 | Guinea Conakry | O. sativa | 0.00 | 0.98 | 0.01 | 0.01 |
| Sorie Kunde | Sierra Leone | O. sativa | 0.00 | 0.98 | 0.01 | 0.01 |
| Chinese red | The Gambia | O. sativa | 0.00 | 0.98 | 0.00 | 0.02 |
| Saidou fire (white grain) | Guinea Conakry | O. sativa | 0.01 | 0.97 | 0.01 | 0.01 |
| Saidou Gbeeli | Guinea Conakry | O. sativa | 0.01 | 0.97 | 0.02 | 0.00 |
| Yaka (Rok3) | Sierra Leone | O. sativa | 0.01 | 0.97 | 0.01 | 0.02 |
| Viotto (off-type?) | Ghana | O. sativa | 0.00 | 0.95 | 0.04 | 0.01 |
| Zomojo | Ghana | O. sativa | 0.01 | 0.95 | 0.04 | 0.01 |
| Zomojo (off-type?) | Ghana | O. sativa | 0.00 | 0.95 | 0.00 | 0.04 |
| Baraso | The Gambia | O. sativa | 0.00 | 0.95 | 0.00 | 0.04 |
| Sarjo Keeba Mano | The Gambia | O. sativa | 0.01 | 0.94 | 0.04 | 0.01 |
| Yaka | Sierra Leone | O. sativa | 0.00 | 0.93 | 0.01 | 0.07 |
| Pa Bad-scent | Sierra Leone | O. sativa | 0.06 | 0.92 | 0.01 | 0.01 |
| Viono short | Ghana | O. sativa | 0.00 | 0.91 | 0.08 | 0.00 |
| Wonyonwonyon yi | Guinea Conakry | O. sativa | 0.02 | 0.89 | 0.04 | 0.05 |
| Terfatch | The Gambia | O. sativa | 0.01 | 0.82 | 0.01 | 0.16 |
| Damansah 1 | Ghana | O. sativa | 0.00 | 0.81 | 0.00 | 0.18 |
| Mani Koyo | Senegal | O. sativa | 0.00 | 0.74 | 0.01 | 0.25 |
| Damansah 4 | Ghana | O. sativa | 0.00 | 0.62 | 0.06 | 0.31 |
| Off-type (in Hombo Wulengo) | The Gambia | O. sativa | 0.00 | 0.61 | 0.00 | 0.38 |
| Bondiyaa Karejang | Senegal | O. sativa | 0.00 | 0.54 | 0.00 | 0.46 |
| Off-type (in Tabuyaa Mani Koyo) | Guinea Bissau | O. sativa | 0.00 | 0.53 | 0.01 | 0.46 |
| Aqua blue | Ghana | O. sativa | 0.00 | 0.48 | 0.44 | 0.07 |
| Aqua blue | Ghana | O. sativa | 0.00 | 0.00 | 1.00 | 0.00 |
| Aqua blue with awns | Ghana | O. sativa | 0.00 | 0.00 | 1.00 | 0.00 |
| Gokpui | Ghana | O. sativa | 0.00 | 0.00 | 1.00 | 0.00 |
| Mateggi | Ghana | O. sativa | 0.00 | 0.00 | 1.00 | 0.00 |
| Buba Njie | Guinea Bissau | O. sativa | 0.00 | 0.00 | 1.00 | 0.00 |
| Bumali | Guinea Bissau | O. sativa | 0.00 | 0.00 | 1.00 | 0.00 |
| Conakry | Guinea Bissau | O. sativa | 0.00 | 0.00 | 1.00 | 0.00 |
| Demba Ba | Guinea Bissau | O. sativa | 0.00 | 0.00 | 1.00 | 0.00 |
| Jahuun (sutungo) | Guinea Bissau | O. sativa | 0.00 | 0.00 | 1.00 | 0.00 |
| Kissidugô | Guinea Bissau | O. sativa | 0.00 | 0.00 | 1.00 | 0.00 |
| Nahawa | Guinea Bissau | O. sativa | 0.00 | 0.00 | 1.00 | 0.00 |
| Off-type (in Sefa Fingo) | Guinea Bissau | O. sativa | 0.00 | 0.00 | 1.00 | 0.00 |
| Sefa Fingo | Guinea Bissau | O. sativa | 0.00 | 0.00 | 1.00 | 0.00 |
| Senkiliba | Guinea Bissau | O. sativa | 0.00 | 0.00 | 1.00 | 0.00 |
| Toba | Guinea Bissau | O. sativa | 0.00 | 0.00 | 1.00 | 0.00 |
| Umobel | Guinea Bissau | O. sativa | 0.00 | 0.00 | 1.00 | 0.00 |
| Usefa Udjenel | Guinea Bissau | O. sativa | 0.00 | 0.00 | 1.00 | 0.00 |
| Conakry | Guinea Conakry | O. sativa | 0.00 | 0.00 | 1.00 | 0.00 |
| Bobordeen | Sierra Leone | O. sativa | 0.00 | 0.00 | 1.00 | 0.00 |
| Boikortor | Sierra Leone | O. sativa | 0.00 | 0.00 | 1.00 | 0.00 |
| Gbengben | Sierra Leone | O. sativa | 0.00 | 0.00 | 1.00 | 0.00 |
| Gbengben | Sierra Leone | O. sativa | 0.00 | 0.00 | 1.00 | 0.00 |
| Jobboi | Sierra Leone | O. sativa | 0.00 | 0.00 | 1.00 | 0.00 |
| Jumukui | Sierra Leone | O. sativa | 0.00 | 0.00 | 1.00 | 0.00 |
| Kondaylah | Sierra Leone | O. sativa | 0.00 | 0.00 | 1.00 | 0.00 |
| Kortigbongoi | Sierra Leone | O. sativa | 0.00 | 0.00 | 1.00 | 0.00 |
| Nduluwai | Sierra Leone | O. sativa | 0.00 | 0.00 | 1.00 | 0.00 |
| Pamanneh | Sierra Leone | O. sativa | 0.00 | 0.00 | 1.00 | 0.00 |
| Pla Gbon | Sierra Leone | O. sativa | 0.00 | 0.00 | 1.00 | 0.00 |
| Sembehun nyaha | Sierra Leone | O. sativa | 0.00 | 0.00 | 1.00 | 0.00 |
| Hombo Wulengo | The Gambia | O. sativa | 0.00 | 0.00 | 1.00 | 0.00 |
| Kukone | The Gambia | O. sativa | 0.00 | 0.00 | 1.00 | 0.00 |
| Kukur | The Gambia | O. sativa | 0.00 | 0.00 | 1.00 | 0.00 |
| Mani Tima | The Gambia | O. sativa | 0.00 | 0.00 | 1.00 | 0.00 |
| Nerica koyo | The Gambia | O. sativa | 0.00 | 0.00 | 1.00 | 0.00 |
| off-type (in Hombo Wulengo) | The Gambia | O. sativa | 0.00 | 0.00 | 1.00 | 0.00 |
| Off-type (in Sefa Koyo) | The Gambia | O. sativa | 0.00 | 0.00 | 1.00 | 0.00 |
| Off-type (Samano?) | The Gambia | O. sativa | 0.00 | 0.00 | 1.00 | 0.00 |
| Sefa Fingo | The Gambia | O. sativa | 0.00 | 0.00 | 1.00 | 0.00 |
| Sefa Fingo (red) | The Gambia | O. sativa | 0.00 | 0.00 | 1.00 | 0.00 |
| Sefa Koyo | The Gambia | O. sativa | 0.00 | 0.00 | 1.00 | 0.00 |
| Sonna Mano | The Gambia | O. sativa | 0.00 | 0.00 | 1.00 | 0.00 |
| Wesiwes | The Gambia | O. sativa | 0.00 | 0.00 | 1.00 | 0.00 |
| Aquablue | Togo | O. sativa | 0.00 | 0.00 | 1.00 | 0.00 |
| Ujogade | Guinea Bissau | O. sativa | 0.01 | 0.00 | 0.99 | 0.00 |
| Uyeey | Guinea Bissau | O. sativa | 0.00 | 0.00 | 0.99 | 0.00 |
| Bonyaha | Sierra Leone | O. sativa | 0.01 | 0.00 | 0.99 | 0.00 |
| Coffeegay.. | Sierra Leone | O. sativa | 0.00 | 0.00 | 0.99 | 0.00 |
| Konowanjei | Sierra Leone | O. sativa | 0.00 | 0.00 | 0.99 | 0.01 |
| Nerica wulengo | The Gambia | O. sativa | 0.00 | 0.00 | 0.99 | 0.00 |
| Sefa Nunfingo | The Gambia | O. sativa | 0.00 | 0.00 | 0.99 | 0.00 |
| Sefa Nunfingo (white) | The Gambia | O. sativa | 0.00 | 0.01 | 0.99 | 0.00 |
| Wab 56-50 | The Gambia | O. sativa | 0.00 | 0.00 | 0.99 | 0.00 |
| Aqua blue | Ghana | O. sativa | 0.00 | 0.00 | 0.98 | 0.01 |
| Off-type (in Kadidjango) | Guinea Bissau | O. sativa | 0.00 | 0.01 | 0.98 | 0.01 |
| Otcha | Guinea Bissau | O. sativa | 0.00 | 0.01 | 0.98 | 0.00 |
| Mabargie | Sierra Leone | O. sativa | 0.00 | 0.01 | 0.98 | 0.01 |
| Yonnie | Sierra Leone | O. sativa | 0.01 | 0.00 | 0.98 | 0.01 |
| Berengdinto Koyo | Guinea Bissau | O. sativa | 0.00 | 0.01 | 0.97 | 0.01 |
| Nerigay | Sierra Leone | O. sativa | 0.00 | 0.02 | 0.97 | 0.01 |
| Yabasie | Sierra Leone | O. sativa | 0.01 | 0.02 | 0.97 | 0.00 |
| Gbengben | Sierra Leone | O. sativa | 0.00 | 0.00 | 0.96 | 0.04 |
| Gbengben | Sierra Leone | O. sativa | 0.03 | 0.00 | 0.96 | 0.01 |
| Musugomie | Sierra Leone | O. sativa | 0.02 | 0.01 | 0.96 | 0.01 |
| Jetteh | Sierra Leone | O. sativa | 0.00 | 0.04 | 0.95 | 0.00 |
| Off-type (lost variety) | The Gambia | O. sativa | 0.00 | 0.02 | 0.95 | 0.02 |
| Jewule | Sierra Leone | O. sativa | 0.04 | 0.01 | 0.94 | 0.02 |
| Konko | Guinea Conakry | O. sativa | 0.00 | 0.07 | 0.93 | 0.00 |
| Ngiligortie | Sierra Leone | O. sativa | 0.05 | 0.02 | 0.93 | 0.00 |
| Red saka | Ghana | O. sativa | 0.02 | 0.00 | 0.91 | 0.06 |
| Off-type (lost variety) | The Gambia | O. sativa | 0.00 | 0.01 | 0.90 | 0.09 |
| Wapu | Guinea Bissau | O. sativa | 0.00 | 0.04 | 0.89 | 0.07 |
| Off-type (in Uyeeye) | Guinea Bissau | O. sativa | 0.00 | 0.06 | 0.87 | 0.07 |
| Kolosarr, original | Guinea Bissau | O. sativa | 0.00 | 0.00 | 0.00 | 1.00 |
| Daakulo Koyo | Senegal | O. sativa | 0.00 | 0.00 | 0.00 | 1.00 |
| Kumoi | The Gambia | O. sativa | 0.00 | 0.00 | 0.00 | 1.00 |
| M Mesengo | The Gambia | O. sativa | 0.00 | 0.00 | 0.00 | 1.00 |
| Mani Wulengo | The Gambia | O. sativa | 0.00 | 0.00 | 0.00 | 1.00 |
| Kolosarr, Bondiya | Guinea Bissau | O. sativa | 0.00 | 0.01 | 0.00 | 0.99 |
| Konsonkuto | Guinea Bissau | O. sativa | 0.00 | 0.00 | 0.00 | 0.99 |
| Maimuna | Guinea Bissau | O. sativa | 0.00 | 0.00 | 0.00 | 0.99 |
| Kissi Foundeyi | Guinea Conakry | O. sativa | 0.00 | 0.00 | 0.00 | 0.99 |
| Wonyonwonyon yi | Guinea Conakry | O. sativa | 0.00 | 0.01 | 0.01 | 0.99 |
| Ablie Mano | Senegal | O. sativa | 0.00 | 0.00 | 0.00 | 0.99 |
| Einu | Senegal | O. sativa | 0.00 | 0.01 | 0.00 | 0.99 |
| Madina Wulengo | Senegal | O. sativa | 0.00 | 0.01 | 0.00 | 0.99 |
| Kari Saba | The Gambia | O. sativa | 0.00 | 0.01 | 0.00 | 0.99 |
| Mani Mesendingo | The Gambia | O. sativa | 0.00 | 0.01 | 0.00 | 0.99 |
| Off-type (in Mani Wulendingo) | The Gambia | O. sativa | 0.00 | 0.01 | 0.00 | 0.99 |
| Kolosarr, M Wulendingo | Guinea Bissau | O. sativa | 0.00 | 0.02 | 0.00 | 0.98 |
| Mesemese | Guinea Bissau | O. sativa | 0.00 | 0.02 | 0.00 | 0.98 |
| Off-type (in Madina Wulengo) | Senegal | O. sativa | 0.00 | 0.02 | 0.01 | 0.98 |
| Binta Sambou | The Gambia | O. sativa | 0.00 | 0.02 | 0.00 | 0.98 |
| Mani Wulendingo | The Gambia | O. sativa | 0.00 | 0.02 | 0.00 | 0.98 |
| Off-type (in Madina Wulengo) | Senegal | O. sativa | 0.00 | 0.01 | 0.03 | 0.95 |
| Kong | Senegal | O. sativa | 0.00 | 0.05 | 0.02 | 0.93 |
| Moti | The Gambia | O. sativa | 0.00 | 0.03 | 0.08 | 0.89 |
| Off-type (in Madina Wulengo) | Senegal | O. sativa | 0.00 | 0.02 | 0.11 | 0.88 |
| Daakulo | Senegal | O. sativa | 0.00 | 0.41 | 0.00 | 0.59 |
| Trimonte | Guinea Conakry | Hybrid | 0.00 | 1.00 | 0.00 | 0.00 |
| Off-type (in Daakulo) | Senegal | Hybrid | 0.00 | 0.99 | 0.00 | 0.00 |
| Ataa | Ghana | Hybrid | 0.00 | 0.71 | 0.00 | 0.28 |
| Off-type (in WAB 56-50) | The Gambia | Hybrid | 0.20 | 0.64 | 0.15 | 0.01 |
| Aquablue awinto | Togo | Hybrid | 0.00 | 0.00 | 1.00 | 0.00 |
| Khaki | Togo | Hybrid | 0.00 | 0.00 | 1.00 | 0.00 |
| Aqua blue signaweh | Ghana | Hybrid | 0.00 | 0.00 | 0.99 | 0.00 |
| Pa Three Month2 | Sierra Leone | Hybrid | 0.00 | 0.01 | 0.99 | 0.01 |
| Nerica 2 (off-type) | Ghana | Hybrid | 0.00 | 0.01 | 0.94 | 0.04 |
| Nerica 2 | Ghana | Hybrid | 0.12 | 0.00 | 0.87 | 0.00 |
| Sewa | Guinea Conakry | Hybrid | 0.00 | 0.13 | 0.86 | 0.00 |
| Off-type (in WAB 56-50) | The Gambia | Hybrid | 0.20 | 0.00 | 0.80 | 0.00 |
| Dissi | Guinea Bissau | Hybrid | 0.00 | 0.00 | 0.00 | 0.99 |
| Jangjango | Guinea Bissau | Hybrid | 0.00 | 0.00 | 0.00 | 0.99 |
| Untufa | Guinea Bissau | Hybrid | 0.00 | 0.00 | 0.01 | 0.99 |
| Wansarang | Guinea Bissau | Hybrid | 0.00 | 0.00 | 0.00 | 0.99 |
| Tebeleh | Sierra Leone | Hybrid | 0.01 | 0.00 | 0.01 | 0.98 |
| Pa Three Month1 | Sierra Leone | Hybrid | 0.00 | 0.05 | 0.01 | 0.95 |
| Pa Three Month3 | Sierra Leone | Hybrid | 0.00 | 0.01 | 0.10 | 0.88 |
| Kaomo with awns | Ghana | unclear | 1.00 | 0.00 | 0.00 | 0.00 |
| Kolonkalan 1b | Sierra Leone | unclear | 1.00 | 0.00 | 0.00 | 0.00 |
| Off-type 1A | Sierra Leone | unclear | 1.00 | 0.00 | 0.00 | 0.00 |
| Pindie | Sierra Leone | unclear | 1.00 | 0.00 | 0.00 | 0.00 |
| Egomu | Ghana | unclear | 0.97 | 0.01 | 0.01 | 0.01 |
| Off-type 1B | Sierra Leone | unclear | 0.96 | 0.02 | 0.00 | 0.02 |
| Pugulu undef. | Ghana | unclear | 0.00 | 0.99 | 0.00 | 0.01 |
| Pugulu white | Ghana | unclear | 0.00 | 0.99 | 0.00 | 0.00 |
| Viono tall | Ghana | unclear | 0.00 | 0.99 | 0.01 | 0.00 |
| Pa Follah | Sierra Leone | unclear | 0.00 | 0.99 | 0.00 | 0.00 |
| Tema | Togo | unclear | 0.00 | 0.97 | 0.00 | 0.03 |
| Pugulu red | Ghana | unclear | 0.00 | 0.95 | 0.02 | 0.03 |
| Pla-Camp | Sierra Leone | unclear | 0.01 | 0.87 | 0.02 | 0.11 |
| Damansah 3 | Ghana | unclear | 0.01 | 0.60 | 0.03 | 0.35 |
| Pugulu undef. | Ghana | unclear | 0.00 | 0.00 | 1.00 | 0.00 |
| Gbondobai | Sierra Leone | unclear | 0.00 | 0.00 | 1.00 | 0.00 |
| Pugulu undef. | Ghana | unclear | 0.00 | 0.00 | 0.99 | 0.00 |
| Jebbeh-komie | Sierra Leone | unclear | 0.01 | 0.00 | 0.98 | 0.00 |
| Bogootie | Sierra Leone | unclear | 0.00 | 0.02 | 0.96 | 0.02 |
| Pindi-pabai 1a red | Sierra Leone | unclear | 0.00 | 0.00 | 0.00 | 1.00 |
| Pa DC | Sierra Leone | unclear | 0.00 | 0.00 | 0.00 | 0.99 |
| Pa Yariken | Sierra Leone | unclear | 0.00 | 0.00 | 0.00 | 0.99 |
| Pa DC | Sierra Leone | unclear | 0.01 | 0.01 | 0.00 | 0.98 |
| Trimont (white) | Sierra Leone | unclear | 0.01 | 0.00 | 0.05 | 0.95 |
|  |  |  |  |  |  |  |
| **B. Modern varieties** |  |  |  |  |  |  |
| I Kong Pao | CIRAD | O. sativa | 0.00 | 1.00 | 0.00 | 0.00 |
| CCA | NARI | O. sativa | 0.00 | 1.00 | 0.00 | 0.00 |
| Parasana | NARI | O. sativa | 0.00 | 1.00 | 0.00 | 0.00 |
| Se 302 G (IRAT 11) | CIRAD | O. sativa | 0.00 | 0.99 | 0.00 | 0.01 |
| Se 319 G (IRAT 12) | CIRAD | O. sativa | 0.00 | 0.99 | 0.00 | 0.01 |
| IR66-23 | IRRI | O. sativa | 0.00 | 0.99 | 0.00 | 0.00 |
| DJ 12-519 | ISRA | O. sativa | 0.00 | 0.99 | 0.00 | 0.00 |
| DJ 8-341 | ISRA | O. sativa | 0.00 | 0.99 | 0.01 | 0.00 |
| Off-type (in DJ-11-307) | NARI | O. sativa | 0.00 | 0.99 | 0.00 | 0.00 |
| RC18-3 | IRRI | O. sativa | 0.00 | 0.98 | 0.01 | 0.01 |
| DJ-11-307 | NARI | O. sativa | 0.00 | 0.97 | 0.00 | 0.03 |
| RC10-43 | IRRI | O. sativa | 0.00 | 0.94 | 0.01 | 0.05 |
| IR36-63 | IRRI | O. sativa | 0.00 | 0.76 | 0.01 | 0.23 |
| IRAT 10 | CIRAD | O. sativa | 0.00 | 0.00 | 1.00 | 0.00 |
| IRAT 110 | WARDA | O. sativa | 0.00 | 0.00 | 1.00 | 0.00 |
| IRAT 112 | WARDA | O. sativa | 0.00 | 0.00 | 1.00 | 0.00 |
| OS 6 (Faro 11) | WARDA | O. sativa | 0.00 | 0.00 | 1.00 | 0.00 |
| WAB 365-B-2-H3-HB | WARDA | O. sativa | 0.00 | 0.00 | 0.99 | 0.00 |
| WAB 450-I-B-P-163-4-1 | WARDA | Hybrid | 0.00 | 0.00 | 1.00 | 0.00 |
| WAB 450-I-B-P-105-HB | WARDA | Hybrid | 0.06 | 0.00 | 0.93 | 0.00 |
| Nerica 1 | MOFA | Hybrid | 0.08 | 0.02 | 0.77 | 0.14 |
|  |  |  |  |  |  |  |
| **C. Wild and semi-wild material** |  |  |  |  |  |  |
| O. barthii black | The Gambia | O. barthii | 1.00 | 0.00 | 0.00 | 0.00 |
| O. barthii white | The Gambia | O. barthii | 1.00 | 0.00 | 0.00 | 0.00 |
| Devil rice | Guinea Conakry | O. barthii | 0.97 | 0.01 | 0.01 | 0.01 |
| Ngafa bei | Sierra Leone | O. barthii | 0.84 | 0.10 | 0.01 | 0.06 |
| Ngewobei | Sierra Leone | O. barthii | 0.75 | 0.19 | 0.02 | 0.04 |
